# Variants of the *IL-10* gene associate with muscle strength in elderly from rural Africa: a candidate gene study

**DOI:** 10.1111/acel.12244

**Published:** 2014-07-18

**Authors:** Karel G M Beenakker, Jacob J E Koopman, David van Bodegom, Maris Kuningas, Pieternella E Slagboom, Johannes J Meij, Andrea B Maier, Rudi G J Westendorp

**Affiliations:** 1 Department of Gerontology and Geriatrics, Leiden University Medical CenterAlbinusdreef 2, Leiden, 2333 ZA, The Netherlands; 2 Leyden Academy on Vitality and AgeingRijnsburgerweg 10, Leiden, 2333 AA, The Netherlands; 3 Department of Epidemiology, Erasmus Medical CenterDr Molewaterplein 50, Rotterdam, 3015 GE, The Netherlands; 4 Department of Medical Statistics, Molecular Epidemiology, Leiden University Medical CenterAlbinusdreef 2, Leiden, 2333 ZA, The Netherlands; 5 Department of Medical Innovation, Amphia HospitalsMolengracht 21, Breda, 4818 CK, The Netherlands; 6 Section of Gerontology and Geriatrics, Department of Internal Medicine, VU University Medical CenterDe Boelelaan 1117, Amsterdam, 1081 HV, The Netherlands

**Keywords:** Africa, cytokine production, *IL-10* gene, innate immunity, muscle strength

## Abstract

Recently, it has been shown that the capacity of the innate immune system to produce cytokines relates to skeletal muscle mass and strength in older persons. The *interleukin-10* (*IL-10*) gene regulates the production capacities of IL-10 and tumour necrosis factor-α (TNF-α). In rural Ghana, *IL-10* gene variants associated with different production capacities of IL-10 and TNF-α are enriched compared with Caucasian populations. In this setting, we explored the association between these gene variants and muscle strength. Among 554 Ghanaians aged 50 years and older, we determined 20 single nucleotide polymorphisms in the *IL-10* gene, production capacities of IL-10 and TNF-α in whole blood upon stimulation with lipopolysaccharide (LPS) and handgrip strength as a proxy for skeletal muscle strength. We distinguished pro-inflammatory haplotypes associated with low IL-10 production capacity and anti-inflammatory haplotypes with high IL-10 production capacity. We found that distinct haplotypes of the *IL-10* gene associated with handgrip strength. A pro-inflammatory haplotype with a population frequency of 43.2% was associated with higher handgrip strength (*P* = 0.015). An anti-inflammatory haplotype with a population frequency of 7.9% was associated with lower handgrip strength (*P* = 0.006). In conclusion, variants of the *IL-10* gene contributing to a pro-inflammatory cytokine response associate with higher muscle strength, whereas those with anti-inflammatory response associate with lower muscle strength. Future research needs to elucidate whether these effects of variation in the *IL-10* gene are exerted directly through its role in the repair of muscle tissue or indirectly through its role in the defence against infectious diseases.

## Introduction

Interleukin-10 (IL-10) is an anti-inflammatory cytokine with important regulatory effects on inflammatory responses. It downregulates the antigen presenting function and inhibits the production of pro-inflammatory cytokines like tumour necrosis factor α (TNF-α) by various immune cells (Hofmann *et al*., 2012). In mice, immune cells producing cytokines are crucial for the repair of skeletal muscle tissue (Arnold *et al*., 2007; Bencze *et al*., 2012; Deng *et al*., 2012; Gao *et al*., 2012). We have recently shown that a higher TNF-α production capacity of immune cells is positively related to muscle mass and muscle strength in a middle-aged Dutch population (Beenakker *et al*., 2013).

The capacity to produce IL-10 and TNF-α upon whole-blood stimulation with lipopolysaccharide (LPS) varies between individuals. This variation is for more than 50% attributable to genetic determinants (Westendorp *et al*., 1997; de Craen *et al*., 2005; Damsgaard *et al*., 2009). The *IL-10* gene is highly polymorphic (Eskdale *et al*., 1998; Kube *et al*., 2001; Kuningas *et al*., 2009), and its haplotypes are transcribed differently (Kurreeman *et al*., 2004). This interindividual variation is extended by variation in the *IL-10* haplotype structure and distribution between ethnicities (Eskdale *et al*., 1998; Moraes *et al*., 2003). We have earlier reported that specific *IL-10* gene variants are enriched in Ghanaian elderly living under adverse conditions (Kuningas *et al*., 2009). These variants have functional significance: some are related to a pro-inflammatory cytokine production capacity, with lower IL-10 and higher TNF-α levels upon whole-blood stimulation with LPS, while others are related to an inverse anti-inflammatory response (Kuningas *et al*., 2009; May *et al*., 2009b; Boef *et al*., 2012). Interestingly, this *IL-10* haplotype structure is less present in Caucasian populations living under affluent conditions (Moraes *et al*., 2003; Kuningas *et al*., 2009), possibly because balanced selection has conserved this haplotype structure in populations under adverse conditions. The functional variation in the genetic determinants of cytokine production capacity forms a meaningful instrument to study the effects of different cytokine production capacities largely free from confounding and reverse causality (Davey Smith & Ebrahim, 2003).

For this study, we had the unique opportunity to study handgrip strength of individuals aged 50 years and older in the Ghanaian population of which we have extensively characterized the *IL-10* gene variants and their effects on cytokine production capacity (Kuningas *et al*., 2009; May *et al*., 2009b; Boef *et al*., 2012). This study aims to investigate the relation between the pro- and anti-inflammatory *IL-10* gene variants, which are not present in Caucasian populations, and handgrip strength as a proxy of overall muscle strength. To account for the possible effects of ill health on handgrip strength, the analyses were also performed after exclusion of subjects with underweight.

## Results

Table 1 displays the characteristics of 554 individuals aged 50 years and older of whom *IL-10* gene variants and handgrip strength were known. Their characteristics were similar as compared with all 4336 individuals of whom *IL-10* gene variants were measured and with all 923 individuals of whom handgrip strength was measured (data not shown). Approximately half of them had a body mass index (BMI) of 18.5 kg m^−2^ or lower, which is regarded as underweight (Shetty & James, 1994; World Health Organization (WHO), 2003). Mean handgrip strength was 27.3 kg (SD 7.6) for those with a normal BMI and 24.1 kg (SD 6.7) for those with underweight. Table S1 shows that handgrip strength did not differ between tribes.

**Table 1:** Characteristics of the study sample (*n* = 554)

Characteristic	Women (*n* = 358)	Men (*n* = 196)
Age, years	63.4 (9.2)	73.0 (9.2)
Tribe, *n* (%)		
Bimoba	252 (70.4)	142 (72.4)
Kusasi	84 (23.5)	44 (22.4)
Mamprusi	12 (3.4)	2 (1.0)
Fulani	1 (0.3)	2 (1.0)
Busanga	8 (2.2)	2 (1.0)
Other or unknown	1 (0.3)	4 (2.0)
Number of households, *n*	299	190
Household property value in US$, median (IQR)	1183 (585–2055)	1028 (580–1782)
Clinical measurements		
Height, cm	157.9 (6.7)	166.0 (6.8)
Weight, kg	45.4 (7.5)	49.4 (7.8)
Body mass index, kg m^−2^	18.2 (2.5)	17.9 (2.3)
Body mass index ≤ 18.5 kg m^−2^, *n* (%)	204 (57.0)	113 (57.6)
Handgrip strength, kg	23.4 (5.9)	29.2 (8.1)

IQR, interquartile range.

Data are presented as means with standard deviations unless otherwise specified.

### IL-10 gene variants and cytokine production capacity

It has been previously shown that several single nucleotide polymorphisms (SNPs) in the *IL-10* gene region influence the production capacities of IL-10 and TNF-α measured in two independent groups in 2006 and 2008 in this research area (Kuningas *et al*., 2009; Boef *et al*., 2012). First, we confirmed that these relations were present in 1177 individuals of whom *IL-10* gene variants were known and of whom measurements of cytokine production capacities were combined from 2005, 2006 and 2008 (Fig. S1A). When restricting this group to the individuals of whom handgrip strength was also known (*n* = 457), a similar pattern was present (Fig. S1B).

It has been previously shown that the SNPs in the *IL-10* gene region constitute two haplotypes that influence the production capacities of IL-10 and TNF-α: a pro-inflammatory haplotype 1 and an anti-inflammatory haplotype 3 (Kuningas *et al*., 2009; Boef *et al*., 2012). We reanalysed the relation between the haplotypes of the *IL-10* gene and cytokine production capacities in the 1177 individuals of whom *IL-10* gene variants were known and of whom measurements of cytokine production capacities were combined from 2005, 2006 and 2008. We confirmed an additive genetic effect for both haplotypes. With each additional copy of the pro-inflammatory haplotype 1, *z*-scores of IL-10 production capacity were 0.08 lower (SE 0.04; *P* for trend = 0.028) and *z*-scores of TNF-α production capacity were 0.11 higher (SE 0.04; *P* for trend = 0.001). With each additional copy of the anti-inflammatory haplotype 3, *z*-scores of IL-10 production capacity were 0.19 higher (SE 0.07; *P* for trend = 0.005) and z-scores of TNF-α were 0.10 lower (SE 0.07; *P* for trend = 0.167). When restricting this group to the individuals of whom handgrip strength was also known (*n* = 457), similar patterns were found (data not shown). Haplotype 2 was not related with the production capacities of IL-10 (*P* for trend = 0.813) or TNF-α (*P* for trend = 0.364) and was used in this study as a negative control. Results were not different between men and women (data not shown).

### IL-10 gene variants and handgrip strength

**Figure 1:**
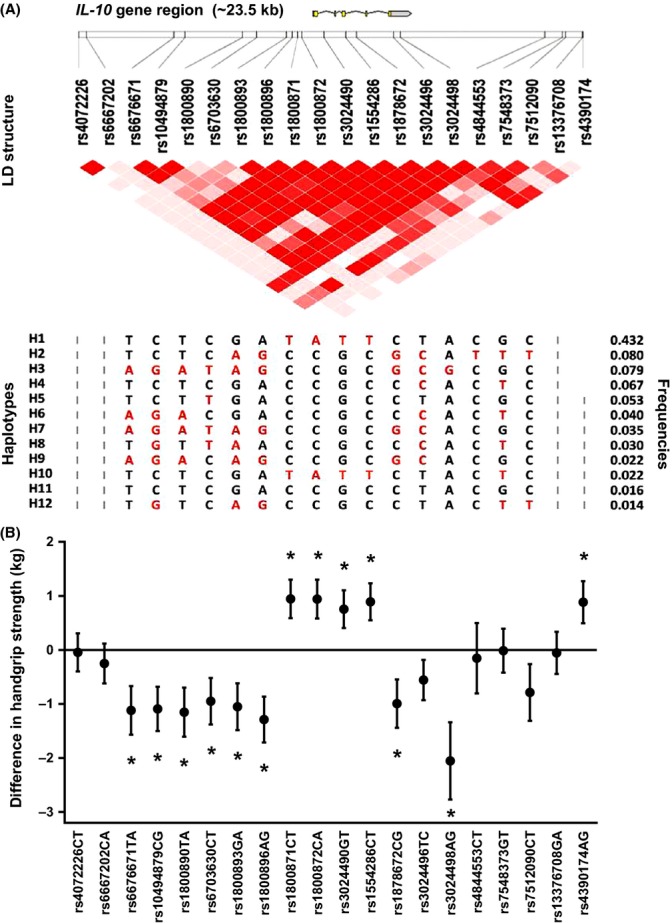
Association of *IL-10* gene SNPs with handgrip strength. (A) A schematic overview of the *IL-10* gene region with the locations of the genotyped single nucleotide polymorphisms (SNPs) indicated by vertical lines. Pairwise linkage disequilibrium (LD) as observed in the entire genotyped population (*n* = 4336) is depicted in red. Population frequencies of the different haplotypes (if > 1%) are presented with the minor alleles of each SNP indicated in red (Kuningas *et al*., 2009). (B) The relation between the minor allele of each *IL-10* gene SNP and handgrip strength for individuals of whom *IL-10* gene variants and handgrip were known (*n* = 554). Handgrip strength is expressed as the deviance from the population’s mean in kilograms (kg) with standard error bars for carriers of at least one copy of the minor allele, adjusted for age, sex, tribe, household and height (*P < 0.05).

Figure 1A shows the twenty genotyped SNPs in the *IL-10* gene region. Most of the SNPs tag a single linkage disequilibrium (LD) block. The haplotype structure and the frequencies of these haplotypes were previously calculated for all individuals of whom *IL-10* gene variants were measured (*n* = 4336) (Kuningas *et al*., 2009). Haplotype frequencies and Hardy–Weinberg equilibriums of the SNPs were not materially different when restricting this group to the individuals of whom handgrip strength was also known (Tables S2 and S3). Furthermore, allelic frequencies were not different between tribes, with a few exceptions between small and large tribes (Table S4).

Figure 1B shows that in the individuals of whom *IL-10* gene variants and handgrip strength were known, carriers of distinct SNPs had higher or lower handgrip strength when compared with noncarriers. The pattern followed the predefined anti-inflammatory and pro-inflammatory haplotype structures shown in Figure 1A and Fig. S1. Results were not different between men and women (data not shown).

**Figure 2:**
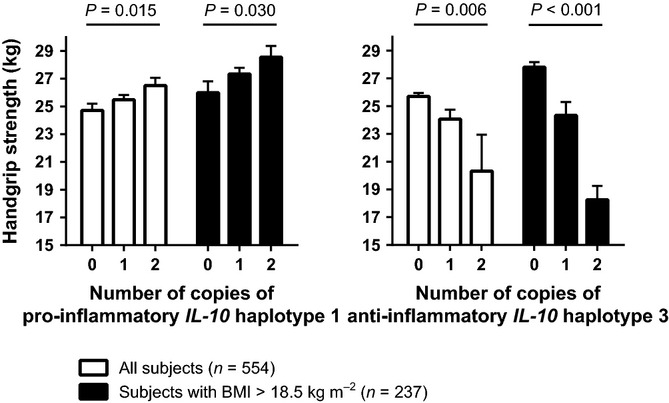
Association of *IL-10* gene haplotypes with handgrip strength in all subjects and in subjects with a normal BMI. Handgrip strength for individuals of whom *IL-10* gene variants and handgrip were known (*n* = 554) presented as means with standard error bars, adjusted for age, sex, tribe, household and height (*P* values for trend). A BMI of 18.5 kg m^−2^ or lower is regarded as underweight (Shetty & James, 1994; World Health Organization (WHO), 2003). For the haplotype structures and frequencies, see Figure 1A.

Figure 2 shows handgrip strength for carriers and noncarriers of the pro-inflammatory haplotype 1 and the anti-inflammatory haplotype 3. We observed an additive genetic effect on handgrip strength, which increased with each additional copy of the pro-inflammatory haplotype 1 and decreased with each additional copy of the anti-inflammatory haplotype 3. Among subjects with a normal BMI, the positive association between the pro-inflammatory haplotype 1 and handgrip strength was equally strong (*P* for interaction = 0.398) and the negative association between the anti-inflammatory haplotype 3 and handgrip strength was stronger (*P* for interaction = 0.009). Both haplotypes were not associated with BMI, neither in all subjects nor in those with BMI above 18.5 kg m^−2^ (*P* for trend > 0.200). Contrary to haplotypes 1 and 3, haplotype 2 was not associated with handgrip strength (*P* for trend = 0.922). Results were not different between men and women (data not shown).

### Cytokine production capacity and handgrip strength

**Figure 3:**
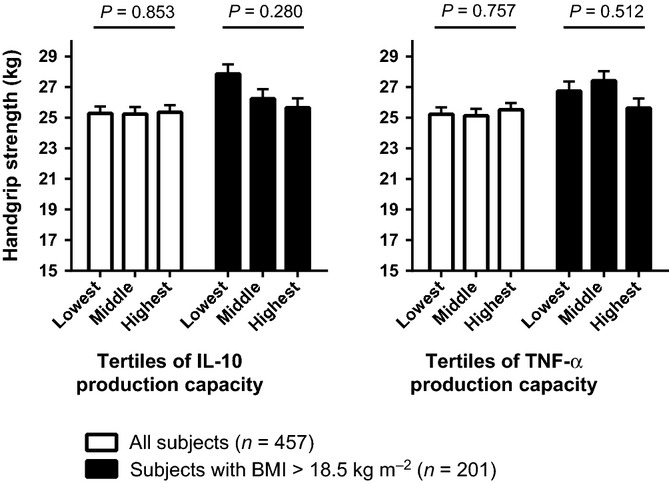
Association of cytokine production capacities with handgrip strength in all subjects and in subjects with a normal BMI. Handgrip strength for individuals of whom *IL-10* gene variants, cytokine production capacities and handgrip strength were known (*n* = 457) presented as means with standard error bars, adjusted for age, sex, tribe, household and height (*P* values for trend). A BMI of 18.5 kg m^−2^ or lower is regarded as underweight (Shetty & James, 1994; World Health Organization (WHO), 2003).

Figure 3 shows that IL-10 and TNF-α production capacities were not related to handgrip strength, although an increase in IL-10 production capacity concurred with a declining trend in handgrip strength among subjects with a normal BMI. When stratifying by sex, IL-10 production capacity was not associated with handgrip strength in either men or women (*P* for trend > 0.800), while TNF-α production capacity was positively associated with handgrip strength in men (*n* = 128; *P* for trend = 0.007) but not in women (*n* = 329; *P* for trend = 0.819).

## Discussion

We found that distinct haplotypes of the *IL-10* gene, associated with variation in the cytokine production capacities of immune cells, were related to handgrip strength in rural Ghana. A pro-inflammatory haplotype with a population frequency of 43.2% was associated with higher handgrip strength, while an anti-inflammatory haplotype with a population frequency of 7.9% was associated with lower handgrip strength. These associations were most outspoken after exclusion of individuals with underweight.

We investigated the effect of *IL-10* gene variants as genetic determinants of the *ex vivo* production capacities of IL-10 and TNF-α by various immune cells in blood (Kuningas *et al*., 2009; Boef *et al*., 2012). The impact of genetic variants on the cytokine production capacity by specific immune cells is not precisely known, but whole-blood stimulation with LPS has been shown to particularly reflect the variance in cytokine production by monocytes (Damsgaard *et al*., 2009). Using genetic determinants, the analyses of the influence of cytokine production capacity on muscle strength are largely free from confounding and reverse causality (Davey Smith & Ebrahim, 2003). This is an advantage compared with our earlier research in which cytokine production capacity was only measured on the phenotypic level (Beenakker *et al*., 2013).

Mice studies have shown that repair and maintenance of skeletal muscle tissue are dependent on the innate immune system (Arnold *et al*., 2007; Bencze *et al*., 2012; Deng *et al*., 2012; Gao *et al*., 2012). Critical for this role of the innate immune system are monocytes infiltrating muscle tissue after injury (Lu *et al*., 2011; Nguyen *et al*., 2011) and producing pro-inflammatory cytokines, of which most notably TNF-α (Warren *et al*., 2002; Chen *et al*., 2005). As a counterbalance, the anti-inflammatory cytokine IL-10 downregulates the pro-inflammatory functioning of monocytes and the production of pro-inflammatory cytokines such as TNF-α (Mosser & Zhang, 2008) and is associated with deceleration of skeletal muscle regeneration (Gao *et al*., 2012). In humans, we have recently reported that a higher capacity to produce pro-inflammatory cytokines, including TNF-α, coexists with higher muscle strength and muscle mass (Beenakker *et al*., 2013). We now show that the same associations exist between genetic determinants of the cytokine production capacity and handgrip strength. These findings support that cytokine production capacity might be important for human muscle repair and maintenance as well.

As an alternative explanation of the association between the *IL-10* gene variants and muscle strength, pro-inflammatory *IL-10* gene variants might yield a better resistance to infectious diseases and thereby a better resistance to muscle wasting due to disease. Cytokine production capacity is related to the incidence and severity of infectious diseases (Mege *et al*., 2006). In rural Ghana, infectious diseases are the main causes of death (Ghana Health Service (GHS), 2005) and we have earlier observed in this research area that carriers of a pro-inflammatory *IL-10* gene haplotype have a survival advantage when drinking from pathogen-rich sources like open wells and rivers (Kuningas *et al*., 2009). Others have found another anti-inflammatory genetic variant associated with a higher IL-10 production capacity to be more prevalent among tuberculosis patients compared with healthy controls in Gambia (Awomoyi *et al*., 2002). Such a mechanism could explain why no relation between a SNP on the *IL-10* gene and handgrip strength was found in a Caucasian population living in an environment where the pathogenic burden is relatively low (Dato *et al*., 2010).

Infectious diseases and malnutrition, which are common in this research area (Ghana Health Service (GHS), 2005; Hesselberg & Yaro, 2006; Ghana Statistical Service (GSS), 2009; Koopman *et al*., 2012; Boef *et al*., 2013), are closely associated with underweight (Shetty & James, 1994). In an attempt to account for the possible effects of ill health on handgrip strength, we repeated the analyses after exclusion of subjects with underweight. Among subjects with a normal BMI, we found an equal relation between the pro-inflammatory haplotype and a stronger relation between the anti-inflammatory haplotype and muscle strength. Moreover, we found that the haplotypes that were associated with handgrip strength were not associated with BMI. These findings indicate that the relation between *IL-10* gene variants and handgrip strength is unlikely to be largely shaped by differences in health.

Although *IL-10* gene variants were related to handgrip strength, the production capacity of IL-10 was not associated with handgrip strength. As a possible explanation, monocytes activated by a pro-inflammatory stimulus like LPS migrate into injured muscle tissue and change only after 2 days into macrophages with an anti-inflammatory phenotype (Arnold *et al*., 2007). We measured the cytokine production capacity of IL-10 24 h after stimulation with LPS, which could have been too early to measure the maximum IL-10 production capacity. In addition, IL-10 is known to have auto-regulatory effects, as it strongly inhibits IL-10 mRNA synthesis in LPS-activated monocytes (de Waal Malefyt *et al*., 1991). This could have diluted the IL-10 production capacity measurement. Another explanation is that the relation might be confounded. Firstly, depletion of muscle tissue by malnutrition or disease could have disrupted the beneficial role of IL-10 production capacity in muscle repair and maintenance. As BMI is likely to reflect muscle mass in this lean population, the stronger relation between *IL-10* gene variants and handgrip strength in the higher BMI stratum points at this possibility. Secondly, infectious diseases might have interfered with our measurements of IL-10 cytokine production capacity. Earlier, we have shown that infectious diseases are highly endemic in the research area and induce a pro-inflammatory immune response (Boef *et al*., 2013). The relation between the *IL-10* gene variants and cytokine production capacity was less outspoken in the smaller group with available data on handgrip strength, possibly due to such environmental factors (May *et al*., 2009a). Thirdly, physical activity attenuates the production capacity of monocytes (Walsh *et al*., 2011) but meanwhile improves muscle strength (Ferreira *et al*., 2012). In this population, physical activity is of vital importance due to the manual labour in farming and housekeeping that is necessary for subsistence up to the highest ages. Fourthly, while we measured cytokine production in whole blood, muscle tissue is recognized to be a cytokine producing organ itself. Although little has been reported about the muscle-specific production of IL-10, the *IL-10* gene might exert its effects in muscle tissue in an autocrine and paracrine manner (Pedersen & Febbraio, 2012). Such a mechanism would not be revealed by the analysis of cytokine production capacity in whole-blood samples.

While we observed no relation between IL-10 production capacity and handgrip strength, we observed a positive relation between TNF-α production capacity and handgrip strength in men, but not in women. This finding is in agreement with our research in Caucasians (Beenakker *et al*., 2013). There we found a men-specific positive relation between TNF-α production capacity upon stimulation with LPS, a Toll-like receptor (TLR) four agonist, and muscle mass and strength. A women-specific positive relation was found between TNF-α production capacity upon stimulation with Pam3Cys-SK4, a TLR-2/1 receptor agonist, and muscle mass. These findings indicate that in skeletal muscle tissue, the TRL-4 pathway is predominant in men and the TLR-2/1 pathway is predominant in women.

Our study has some limitations. Firstly, as in all genetic association studies, we cannot exclude that the *IL-10* gene variants are in linkage disequilibrium with variants of other genes that affect handgrip strength. However, we have previously described that this is unlikely, because resequencing of the *IL-10* gene region and its surroundings did not result in any variants additional to the SNPs that were genotyped in the *IL-10* gene (Kuningas *et al*., 2009). Secondly, we did not document possible epigenetic variation in the *IL-10* gene. There is growing evidence that caloric intake and dietary composition modify epigenetic marks (Li *et al*., 2011; McKay & Mathers, 2011), which can influence transcription of the *IL-10* gene in immune cells (Villagra *et al*., 2010). Malnutrition, being common in the research area (Ghana Health Service (GHS), 2005; Hesselberg & Yaro, 2006; Ghana Statistical Service (GSS), 2009; Koopman *et al*., 2012), could thereby affect the relation between the *IL-10* gene and muscle strength. Lastly, this is a cross-sectional study, while it would be valuable to associate *IL-10* gene variants with longitudinal decline in handgrip strength over age.

In conclusion, this study shows that *IL-10* gene variants associate with the production capacities of IL-10 and TNF-α and strongly relate to handgrip strength in rural Africa. A haplotype reflecting a pro-inflammatory immune response was associated with higher muscle strength, while a haplotype reflecting an anti-inflammatory immune response was associated with lower muscle strength, especially after exclusion of individuals with underweight. Future studies are needed to elucidate whether variants of the *IL-10* gene determine handgrip strength through their role in the repair of skeletal muscle tissue directly or indirectly through their role in the defence against infectious diseases.

## Methods

### Research area

This study was performed in a remote, rural and underdeveloped area in the Upper East Region in Ghana in West Africa. The vast majority of the inhabitants are involved in noncommercial agriculture performed by manual labour. Infectious diseases are the main causes of death (Ghana Health Service (GHS), 2005). The prevalence of human immunodeficiency virus (HIV) is low (< 4%) compared with other sub-Saharan regions (Ghana Health Service (GHS), 2005). Since 2002, we have followed a horticultural population in the Garu-Tempane District in the Upper East Region, comprising approximately 25 000 inhabitants living in circa 40 villages. For each household, we determined the household property value and the socioeconomic status in 2007 according to the Demographic and Health Survey method (van Bodegom *et al*., 2009). Elaborate descriptions of the research population have been given elsewhere (Meij *et al*., 2007; van Bodegom *et al*., 2009; Kuningas *et al*., 2009; Koopman *et al*., 2012).

### Ethical approval

Ethical approval was given by the Ethical Review Committee of Ghana Health Services, the Committee Medical Ethics of the Leiden University Medical Center, and by the local chiefs and elders. Because of illiteracy, informed consent was obtained orally from the participants in the local language. A consent form was read out to each participant with an explanation on the purpose and conduction of this research project.

### DNA collection and genotyping

We collected buccal swabs between 2002 and 2006 for 4336 individuals (Kuningas *et al*., 2009). Common genetic variation (minor allele frequency ≥ 5%) in the *IL-10* gene region was determined by genotyping 20 SNPs, selected from the Yoruba population in the HapMap database (release #21, *r*^2^ = 0.8) and genotyped using mass spectrometry (Seqeunom Inc, San Diego, USA). All SNPs were in Hardy–Weinberg equilibrium, with one exception where a minor deviation was observed (Kuningas *et al*., 2009). We have recently reported that population stratification is unlikely to influence associations with genetic variation in autosomal genes, as analysis of autosomal DNA, mtDNA and y-chromosomal genetic variation patterns in the research area revealed that female-mediated gene flow is nearly fully random, whereas male-mediated gene flow is highly reduced (Sanchez-Faddeev *et al*., 2013). This genetic substructure is an immediate result of the patrilocal society. We addressed residual population stratification by adjusting all analyses for tribe. Familial relatedness among individuals was addressed by adjusting all analyses for household.

### Handgrip strength and BMI

Handgrip strength and BMI were measured in 2009 and 2010 in 923 individuals aged 50 years and older, recruited independently from the genetic samples. Data on the *IL-10* gene were available for 554 of them. We consecutively visited all villages in the research area, in which we set up a mobile fieldwork station. We approached all individuals aged 50 years and older and brought less mobile participants by car. Inclusion was limited by the duration of both field visits. Individuals did not participate if they were unable to leave the house, were absent from the research area for a longer period or refused participation. Handgrip strength was measured using a calibrated Jamar hand dynamometer (Sammons Preston Inc., Bolingbrook, IL) with the participant in an upright position and the arm of the measured hand unsupported parallel to the body. The width of the dynamometer’s handle was adjusted to each participant’s hand size so that the middle phalanges rested on the inner handle. The participants were instructed to exert maximal force once by each hand. We used the highest measurement of both hands in our analyses. Body height and weight were measured by a calibrated length scale and weighing scale. A BMI of 18.5 kg m^−2^ or lower was defined as underweight and a BMI above 18.5 kg m^−2^ as a normal BMI, according to the classification of the Food and Agriculture Organization and World Health Organization (Shetty & James, 1994; World Health Organization (WHO), 2003).

### Cytokine production capacity

The production capacities of IL-10 and TNF-α were measured in blood samples that were taken in 2005, 2006 or 2008. Previous publications, studying measurements from these years separately, have reported the procedure by which the blood samples were processed (May *et al*., 2009a,b; Boef *et al*., 2012). Venous blood was locally incubated with *Escherichia coli* LPS, the supernatants frozen and shipped for measurement of cytokine concentrations in the Netherlands by enzyme-linked immunosorbent assay (ELISA). The procedure has been reported to have a small intra-individual compared with the interindividual variation (Damsgaard *et al*., 2009) and to be replicable with an interval of 2 years in this research area (May *et al*., 2009a; Boef *et al*., 2012). We combined the measurements from all 3 years of 1177 individuals of whom data on the *IL-10* gene were also available.

### Statistical analyses

The program Haploview (Barrett *et al*., 2005) was used to test for Hardy–Weinberg equilibrium. Statistical analyses were performed with IBM SPSS Statistics version 20 and StataCorp Stata/SE version 12.0; College Station, TX, USA. The relations between *IL-10* gene SNPs and haplotype copies, cytokine production capacities, and handgrip strength were assessed by linear mixed models. These analyses were adjusted for age, sex and tribe as fixed factors and household as a random factor. Analyses with handgrip strength were additionally adjusted for height as a fixed factor. Cytokine concentrations were natural-logarithmically transformed due to skewedness and standardized as z-scores ((individual level − mean level)/standard deviation) per sex (Aulock *et al*., 2006) within each year of measurement. The z-scores of 2005, 2006 and 2008 were averaged into one z-score per individual. Haplotypes were defined as pro-inflammatory if associated with lower levels of IL-10 and higher levels of TNF-α upon stimulation with LPS. Haplotypes were defined as anti-inflammatory if associated with higher levels of IL-10 and lower levels of TNF-α upon stimulation with LPS (Kuningas *et al*., 2009). In all haplotype analyses, the posterior probabilities of pairs of haplotypes per individual, as estimated by PHASE (Stephens *et al*., 2001), were used as weights.

## Supporting Information

**Fig. S1** Association of *IL-10* gene SNPs with cytokine production capacities. The relation between the minor allele of each *IL-10* gene SNP and the production capacities of IL-10 and TNF-α for (A) individuals of whom *IL-10* gene variants and cytokine production capacities were known (*n* = 1177) and (B) individuals of whom *IL-10* gene variants, cytokine production capacities and handgrip strength were known (*n* = 457). Cytokine production capacities are expressed as z-scores with standard error bars for carriers of at least one copy of the minor allele, adjusted for age, sex, tribe and household (**P* < 0.05).

**Table S1** Association between tribe and handgrip strength. **Table S2** Haplotype frequencies in the study population and in the entire genotyped population. **Table S3** Locations and minor allele frequencies of the *IL10* gene SNPs in the study population and in the entire genotyped population. **Table S4** Minor allele frequencies of *IL-10* gene SNPs for the included tribes.

## References

[b1] Arnold L, Henry A, Poron F, Baba-Amer Y, van Rooijen N, Plonquet A, Gherardi RK, Chazaud B (2007). Inflammatory monocytes recruited after skeletal muscle injury switch into antiinflammatory macrophages to support myogenesis. J. Exp. Med.

[b2] Aulock SV, Deininger S, Draing C, Gueinzius K, Dehus O, Hermann C (2006). Gender difference in cytokine secretion on immune stimulation with LPS and LTA. J. Interferon Cytokine Res.

[b3] Awomoyi AA, Marchant A, Howson JM, McAdam KP, Blackwell JM, Newport MJ (2002). Interleukin-10, polymorphism in SLC11A1 (formerly NRAMP1), and susceptibility to tuberculosis. J. Infect. Dis.

[b4] Barrett JC, Fry B, Maller J, Daly MJ (2005). Haploview: analysis and visualization of LD and haplotype maps. Bioinformatics.

[b5] Beenakker KG, Westendorp RG, de Craen AJ, Slagboom PE, van Heemst D, Maier AB (2013). Pro-inflammatory capacity of classically activated monocytes relates positively to muscle mass and strength. Aging Cell.

[b6] Bencze M, Negroni E, Vallese D, Yacoub-Youssef H, Chaouch S, Wolff A, Aamiri A, Di Santo JP, Chazaud B, Butler-Browne G, Savino W, Mouly V, Riederer I (2012). Proinflammatory macrophages enhance the regenerative capacity of human myoblasts by modifying their kinetics of proliferation and differentiation. Mol. Ther.

[b7] van Bodegom D, May L, Kuningas M, Kaptijn R, Thomese F, Meij HJ, Amankwa J, Westendorp RG (2009). Socio-economic status by rapid appraisal is highly correlated with mortality risks in rural Africa. Trans. R. Soc. Trop. Med. Hyg.

[b8] Boef AG, May L, van Bodegom D, Kuningas M, Eriksson UK, Westendorp RG (2012). The influence of genetic variation on innate immune activation in an environment with high infectious pressure. Genes Immun.

[b9] Boef AG, May L, van Bodegom D, van Lieshout L, Verweij JJ, Maier AB, Westendorp RG, Eriksson UK (2013). Parasitic infections and immune function: effect of helminth infections in a malaria endemic area. Immunobiology.

[b10] Chen SE, Gerken E, Zhang Y, Zhan M, Mohan RK, Li AS, Reid MB, Li YP (2005). Role of TNF-α signaling in regeneration of cardiotoxin-injured muscle. Am. J. Physiol. Cell Physiol.

[b11] de Craen AJ, Posthuma D, Remarque EJ, van den Biggelaar AH, Westendorp RG, Boomsma DI (2005). Heritability estimates of innate immunity: an extended twin study. Genes Immun.

[b12] Damsgaard CT, Lauritzen L, Calder PC, Kjær TM, Frøkiær H (2009). Whole-blood culture is a valid low-cost method to measure monocytic cytokines: a comparison of cytokine production in cultures of human whole-blood, mononuclear cells and monocytes. J. Immunol. Methods.

[b13] Dato S, Krabbe KS, Thinggaard M, Pedersen BK, Christensen K, Bruunsgaard H, Christiansen L (2010). Commonly studied polymorphisms in inflammatory cytokine genes show only minor effects on mortality and related risk factors in nonagenarians. J. Gerontol. A Biol. Sci. Med. Sci.

[b14] Davey Smith G, Ebrahim S (2003). ‘Mendelian randomization’: can genetic epidemiology contribute to understanding environmental determinants of disease?. Int. J. Epidemiol.

[b15] Deng B, Wehling-Henricks M, Villalta SA, Wang Y, Tidball JG (2012). IL-10 triggers changes in macrophage phenotype that promote muscle growth and regeneration. J. Immunol.

[b16] Eskdale J, Gallagher G, Verweij CL, Keijsers V, Westendorp RG, Huizinga TW (1998). Interleukin 10 secretion in relation to human IL-10 locus haplotypes. Proc. Natl Acad. Sci. USA.

[b17] Ferreira ML, Sherrington C, Smith K, Carswell P, Bell R, Bell M, Nascimento DP, Máximo Pereira LS, Vardon P (2012). Physical activity improves strength, balance and endurance in adults aged 40–65 years: a systematic review. J. Physiother.

[b18] Gao Y, Li Y, Guo X, Wu Z, Zhang W (2012). Loss of STAT1 in bone marrow-derived cells accelerates skeletal muscle regeneration. PLoS ONE.

[b19] Ghana Health Service (GHS) (2005). Annual Report 2004 Upper East Regional Health Administration.

[b20] Ghana Statistical Service (GSS) (2009). Ghana Demographic and Health Survey 2008.

[b21] Hesselberg J, Yaro JA (2006). An assessment of the extent and causes of food insecurity in northern Ghana using a livelihood vulnerability framework. GeoJournal.

[b22] Hofmann SR, Rösen-Wolff A, Tsokos GC, Hedrich CM (2012). Biological properties and regulation of IL-10 related cytokines and their contribution to autoimmune disease and tissue injury. Clin. Immunol.

[b23] Koopman JJ, van Bodegom D, Jukema JW, Westendorp RG (2012). Risk of cardiovascular disease in a traditional african population with a high infectious load: a population-based study. PLoS ONE.

[b24] Kube D, Rieth H, Eskdale J, Kremsner PG, Gallagher G (2001). Structural characterisation of the distal 5′ flanking region of the human interleukin-10 gene. Genes Immun.

[b25] Kuningas M, May L, Tamm R, van Bodegom D, van den Biggelaar AH, Meij JJ, Frölich M, Ziem JB, Suchiman HE, Metspalu A, Slagboom PE, Westendorp RG (2009). Selection for genetic variation inducing pro-inflammatory responses under adverse environmental conditions in a Ghanaian population. PLoS ONE.

[b26] Kurreeman FA, Schonkeren JJ, Heijmans BT, Toes RE, Huizinga TW (2004). Transcription of the IL10 gene reveals allele-specific regulation at the mRNA level. Hum. Mol. Genet.

[b27] Li Y, Daniel M, Tollefsbol TO (2011). Epigenetic regulation of caloric restriction in aging. BMC Med.

[b28] Lu H, Huang D, Ransohoff RM, Zhou L (2011). Acute skeletal muscle injury: CCL2 expression by both monocytes and injured muscle is required for repair. FASEB J.

[b29] May L, van Bodegom D, Kuningas M, Meij JJ, de Craen AJ, Frölich M, Westendorp RG (2009a). Performance of the whole-blood stimulation assay for assessing innate immune activation under field conditions. Cytokine.

[b30] May L, van den Biggelaar AH, van Bodegom D, Meij HJ, de Craen AJ, Amankwa J, Frölich M, Kuningas M, Westendorp RG (2009b). Adverse environmental conditions influence age-related innate immune responsiveness. Immun. Ageing.

[b31] McKay JA, Mathers JC (2011). Diet induced epigenetic changes and their implications for health. Acta Physiol. (Oxf.).

[b32] Mege JL, Meghari S, Honstettre A, Capo C, Raoult D (2006). The two faces of interleukin 10 in human infectious diseases. Lancet Infect. Dis.

[b33] Meij JJ, Van Bodegom D, Laar B, Meij JJ (2007). The Bimoba: the people of Yennu. Testing Life History Theory in a Contemporary African Population.

[b34] Moraes MO, Santos AR, Schonkeren JJ, Vanderborght PR, Ottenhoff TH, Moraes ME, Moraes JR, Sampaio EP, Sarno EN, Huizinga TW (2003). Interleukin-10 promoter haplotypes are differently distributed in the Brazilian versus the Dutch population. Immunogenetics.

[b135] Mosser DM, Zhang X (2008). Interleukin-10: new perspectives on an old cytokine. Immunol. Rev.

[b35] Nguyen M-H, Cheng M, Koh TJ (2011). Impaired muscle regeneration in ob/ob and db/db mice. ScientificWorldJournal.

[b36] Pedersen BK, Febbraio MA (2012). Muscles, exercise and obesity: skeletal muscle as a secretory organ. Nature Rev. Endocrinol.

[b37] Sanchez-Faddeev H, Pijpe J, van der Hulle T, Meij HJ, JvdG K, Slagboom PE, Westendorp RG, de Knijff P (2013). The influence of clan structure on the genetic variation in a single Ghanaian village. Eur. J. Hum. Genet.

[b38] Shetty PS, James WPT (1994). Body mass index: a measure of chronic energy deficiency in adults. FAO Food Nutr. Pap.

[b39] Stephens M, Smith NJ, Donnelly P (2001). A new statistical method for haplotype reconstruction from population data. Am. J. Hum. Genet.

[b40] Villagra A, Sotomayor EM, Seto E (2010). Histone deacetylases and the immunological network: implications in cancer and inflammation. Oncogene.

[b41] de Waal Malefyt R, Abrams J, Bennett B, Figdor CG, de Vries JE (1991). Interleukin 10 (IL-10) inhibits cytokine synthesis by human monocytes: an autoregulatory role of IL-10 produced by monocytes. J. Exp. Med.

[b42] Walsh NP, Gleeson M, Shephard RJ, Gleeson M, Woods JA, Bishop NC, Fleshner M, Green C, Pedersen BK, Hoffman-Goetz L, Rogers CJ, Northoff H, Abbasi A, Simon P (2011). Position statement. Part one: immune function and exercise. Exerc. Immunol. Rev.

[b43] Warren GL, Hulderman T, Jensen N, McKinstry M, Mishra M, Luster MI, Simeonova PP (2002). Physiological role of tumor necrosis factor alpha in traumatic muscle injury. FASEB J.

[b44] Westendorp RG, Langermans JA, Huizinga TW, Elouali AH, Verweij CL, Boomsma DI, Vandenbroucke JP (1997). Genetic influence on cytokine production and fatal meningococcal disease. Lancet.

[b45] World Health Organization (WHO) (2003). Diet, nutrition and the prevention of chronic diseases. World Health Organ. Tech. Rep. Ser.

