# Supplementary figures and images for: Variants of the IL-10 gene associate with muscle strength in elderly from rural Africa: a candidate gene study
Source: Aging Cell. 2014 Jul 18;13(5):862–8. doi: 10.1111/acel.12244 (PMC4331746; doi:10.1111/acel.12244)

**A**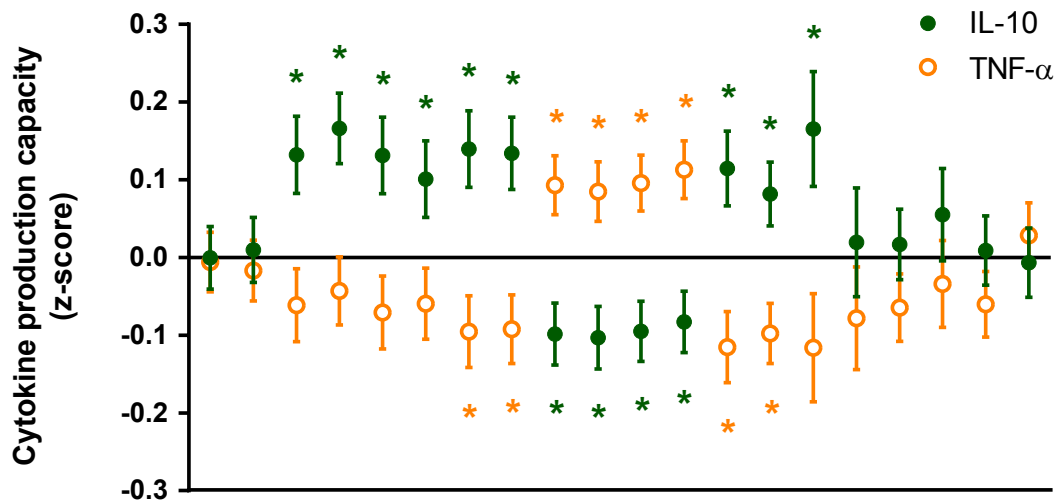**B**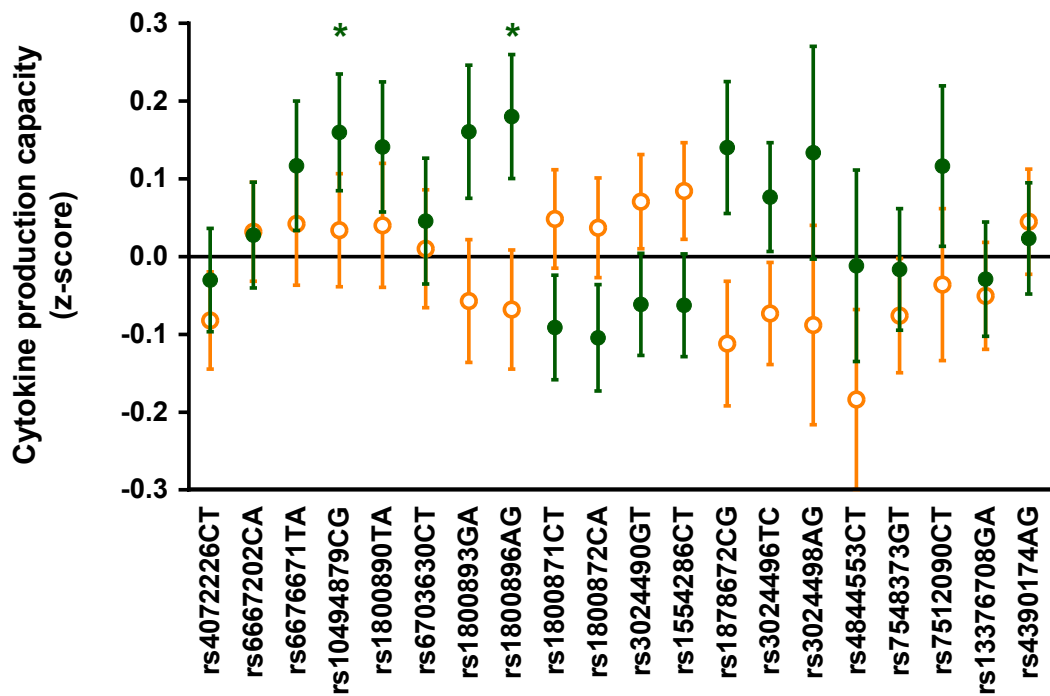

Supplement: Supplementary file 1 — Fig. S1 Association of IL-10 gene SNPs with cytokine production capacities. The relation between the minor allele of each IL-10 gene SNP and the production capacities of IL-10 and TNF-α for (A) individuals of whom IL-10 gene variants and cytokine production capacities were known (n = 1177) and (B) individuals of whom IL-10 gene variants, cytokine production capacities and handgrip strength were known (n = 457). Cytokine production capacities are expressed as z-scores with standard error bars for carriers of at least one copy of the minor allele, adjusted for age, sex, tribe and household (*P < 0.05). [file acel0013-0862-sd1.pdf]
