# Supplementary material for: Variants of the IL-10 gene associate with muscle strength in elderly from rural Africa: a candidate gene study
Source: Aging Cell. 2014 Jul 18;13(5):862–8. doi: 10.1111/acel.12244 (PMC4331746; doi:10.1111/acel.12244)
Supplement: Supplementary file 2 — Table S1 Association between tribe and handgrip strength. Table S2 Haplotype frequencies in the study population and in the entire genotyped population. Table S3 Locations and minor allele frequencies of the IL10 gene SNPs in the study population and in the entire genotyped population. Table S4 Minor allele frequencies of IL-10 gene SNPs for the included tribes. [file acel0013-0862-sd2.docx]

**SUPPLEMENTARY TABLES**

**Supplementary Table S1: Association between tribe and handgrip strength**

|  | **Bimoba** | **Kusasi** | **Mamprusi** | **Fulani** | **Busanga** | **Other** |  |
| --- | --- | --- | --- | --- | --- | --- | --- |
|  | **(*n* = 394)** | **(*n* = 128)** | **(*n* = 14)** | **(*n* = 3)** | **(*n* = 10)** | **(*n* = 3)** | ***p*** |
| Handgrip strength, kg | 26.6 (0.3) | 26.8 (0.6) | 26.3 (1.7) | 21.9 (3.6) | 30.7 (2.0) | 27.7 (2.8) | 0.30 |

Handgrip strength is given as mean with standard error. Values have been adjusted for age and sex. Difference between the tribes has been tested by ANCOVA.

**Supplementary Table S2: Haplotype frequencies in the study population and in the entire genotyped population**

|  | **Haplotype frequencies** | |
| --- | --- | --- |
|  | **Study population** | **Entire genotyped population** |
| **Haplotype** | **(n = 554)** | **(n = 4336)** |
| H1 | 0.457 | 0.432 |
| H2 | 0.066 | 0.080 |
| H3 | 0.063 | 0.079 |
| H4 | 0.069 | 0.067 |
| H5 | 0.048 | 0.053 |
| H6 | 0.040 | 0.040 |
| H7 | 0.048 | 0.035 |
| H8 | 0.021 | 0.030 |
| H9 | 0.026 | 0.022 |
| H10 | 0.021 | 0.022 |
| H11 | 0.012 | 0.016 |
| H12 | 0.015 | 0.014 |

Differences in haplotype frequencies between both populations have been tested by the Pearson chi-squared test (*p* = 0.79).

**Supplementary Table S3: Locations and minor allele frequencies of the *IL10* gene SNPs in the study population and in the entire genotyped population**

|  |  |  | **Study population** | |  | **Entire genotyped** | |
| --- | --- | --- | --- | --- | --- | --- | --- |
|  |  |  |  | |  | **population** | |
|  |  |  | **(*n* = 554)** | |  | **(*n* = 4336)** | |
| ***IL-10* SNPs** | **Alleles^a^** | **Location** | **MAF** | **HWE** |  | **MAF** | **HWE** |
| rs4072226 | C/T | promoter | 0.463 | 0.936 |  | 0.456 | 0.865 |
| rs6667202 | C/A | promoter | 0.499 | 0.851 |  | 0.484 | 0.602 |
| rs6676671 | T/A | promoter | 0.195 | 0.870 |  | 0.200 | 0.832 |
| rs10494879 | C/G | promoter | 0.269 | 0.823 |  | 0.284 | 0.025 |
| rs1800890 | T/A | promoter | 0.192 | 0.822 |  | 0.201 | 0.865 |
| rs6703630 | C/T | promoter | 0.206 | 0.579 |  | 0.220 | 0.066 |
| rs1800893 | G/A | promoter | 0.256 | 0.005 |  | 0.284 | 0.196 |
| rs1800896 | A/G | promoter | 0.260 | 0.064 |  | 0.284 | 0.401 |
| rs1800871 | C/T | promoter | 0.509 | 0.984 |  | 0.470 | 0.302 |
| rs1800872 | C/A | promoter | 0.506 | 0.811 |  | 0.472 | 0.034 |
| rs3024490 | G/T | intron | 0.523 | 0.695 |  | 0.484 | 0.013 |
| rs1554286 | C/T | e/i boundary | 0.500 | 0.433 |  | 0.468 | 0.157 |
| rs1878672 | C/G | intron | 0.228 | 0.072 |  | 0.244 | 0.612 |
| rs3024496 | T/C | exon | 0.393 | 0.928 |  | 0.425 | 0.043 |
| rs3024498 | A/G | exon | 0.066 | 0.904 |  | 0.083 | 0.129 |
| rs4844553 | C/T | 3’ UTR | 0.080 | 0.963 |  | 0.096 | 0.084 |
| rs7548373 | G/T | 3’ UTR | 0.282 | 0.790 |  | 0.297 | 0.190 |
| rs7512090 | C/T | 3’ UTR | 0.126 | 0.681 |  | 0.132 | 0.084 |
| rs13376708 | G/A | 3’ UTR | 0.320 | 0.784 |  | 0.327 | 0.273 |
| rs4390174 | A/G | 3’ UTR | 0.290 | 0.981 |  | 0.282 | 0.630 |

^a^ Major/minor allele. e/i boundary: exon/intron boundary. MAF: minor allele frequency. HWE: *p*-values for Hardy-Weinberg equilibrium.

**Supplementary Table S4: Minor allele frequencies of *IL-10* gene SNPs for the included tribes**

|  | **Minor allele frequencies** | | | | | |  |
| --- | --- | --- | --- | --- | --- | --- | --- |
|  | **Bimoba** | **Kusasi** | **Mamprusi** | **Fulani** | **Busanga** | **Other** |  |
| ***IL-10* SNPs** | **(n=394)** | **(*n* = 128)** | **(*n* = 14)** | **(*n* = 3)** | **(*n* = 10)** | **(*n* = 3)** | ***p*** |
| rs4072226 | 0.443 | 0.516 | 0.536 | 0.500 | 0.400 | 0.500 | 0.29 |
| rs6667202 | 0.524 | 0.429 | 0.464 | 0.250 | 0.550 | 0.400 | 0.10 |
| rs6676671 | 0.186 | 0.213 | 0.250 | 0.000 | 0.300 | 0.200 | 0.25 |
| rs10494879 | 0.367 | 0.276 | 0.286 | 0.167 | 0.350 | 0.300 | 0.48 |
| rs1800890 | 0.183 | 0.211 | 0.250 | 0.000 | 0.300 | 0.125 | 0.33 |
| rs6703630 | 0.194 | 0.238 | 0.250 | 0.000 | 0.300 | 0.300 | 0.12 |
| rs1800893 | 0.261 | 0.262 | 0.154 | 0.167 | 0.222 | 0.000 | 0.20 |
| rs1800896 | 0.260 | 0.264 | 0.231 | 0.177 | 0.300 | 0.200 | 0.87 |
| rs1800871 | 0.509 | 0.508 | 0.429 | 0.833 | 0.550 | 0.500 | 0.79 |
| rs1800872 | 0.506 | 0.504 | 0.429 | 0.833 | 0.550 | 0.500 | 0.77 |
| rs3024490 | 0.527 | 0.512 | 0.429 | 0.833 | 0.556 | 0.500 | 0.92 |
| rs1554286 | 0.503 | 0.500 | 0.364 | 0.833 | 0.500 | 0.333 | 0.71 |
| rs1878672 | 0.227 | 0.238 | 0.154 | 0.167 | 0.462 | 0.100 | 0.80 |
| rs3024496 | 0.398 | 0.399 | 0.423 | 0.177 | 0.350 | 0.250 | 0.34 |
| rs3024498 | 0.059 | 0.089 | 0.071 | 0.000 | 0.001 | 0.000 | 0.56 |
| rs4844553 | 0.087 | 0.075 | 0.036 | 0.000 | 0.000 | 0.000 | 0.044 |
| rs7548373 | 0.300 | 0.258 | 0.250 | 0.000 | 0.056 | 0.200 | 0.009 |
| rs7512090 | 0.141 | 0.105 | 0.105 | 0.038 | 0.000 | 0.000 | 0.003 |
| rs13376708 | 0.308 | 0.352 | 0.357 | 0.167 | 0.250 | 0.600 | 0.23 |
| rs4390174 | 0.275 | 0.321 | 0.321 | 0.667 | 0.350 | 0.200 | 0.19 |

Differences in minor allele frequencies between tribes have been tested by the linear-by-linear association test.
